# Childhood-Onset Lupus Nephritis in the Era of Triple Therapy and Steroid Minimization

**DOI:** 10.3390/children13070930

**Published:** 2026-07-15

**Authors:** Mohamed S. Al Riyami, Badria Al Ghaithi, Sulaiman Al Saidi, Anwar Al Omairi, Naifain Al Kalbani, Naji Al Dhawi

**Affiliations:** 1 Pediatric Nephrology Unit, Department of Child Health, Royal Hospital, Muscat PC 111, Oman; badria.al-ghaithi@moh.gov.om (B.A.G.); suleiman.alsaidi@moh.gov.om (S.A.S.); naifain.alkalbani@moh.gov.om (N.A.K.); 2 Pediatric Nephrology Unit, Department of Child Health, Sultan Qaboos University Hospital, Muscat H5QC+36M, Oman; alomairi@squ.edu.om (A.A.O.); n.alhdawi@squ.edu.om (N.A.D.)

**Keywords:** childhood-onset lupus nephritis, pediatric systemic lupus erythematosus, belimumab, voclosporin, obinutuzumab, mycophenolate, glucocorticoid minimization, triple therapy, kidney biopsy, transition care

## Abstract

Childhood-onset lupus nephritis (cLN) should no longer be framed as a smaller version of adult lupus nephritis. It is a high-stakes pediatric kidney disease in which immune injury, treatment toxicity, growth, puberty, fertility, adherence, and transition to adult care intersect over decades. Approximately 10–20% of systemic lupus erythematosus begins in childhood, and 40–60% of affected children develop lupus nephritis. Regional cohorts report even higher renal involvement in some populations, including 65–73% among Saudi children with SLE. Contemporary guidance has moved from prolonged high-dose glucocorticoids and cyclophosphamide-dominant treatment toward biopsy-driven, treat-to-target care built around mycophenolic acid analogues, hydroxychloroquine, nephroprotection, rapid steroid tapering, and selected belimumab- or calcineurin-inhibitor-based triple therapy. This Viewpoint argues that the central question in cLN is no longer simply how to induce remission, but how to produce a durable kidney response early enough, with sufficiently low cumulative toxicity, to preserve kidney function and childhood development. Pediatric practice must therefore combine adult trial evidence with child-specific caution, explicit adherence strategies, fertility and growth protection, and structured transition planning. The aim should be efficacy without toxicity: not undertreatment, but intelligent, sustainable, developmentally informed treatment.

## 1. Introduction

Childhood-onset systemic lupus erythematosus (SLE) represents a minority of all SLE, but it carries a disproportionate burden of organ-threatening disease. Lupus nephritis is the most important determinant of this burden. Children may present during puberty, often with severe multiorgan activity, frequent relapse, and a lower probability of sustained remission than adults [1]. The clinical problem is therefore not only the acute nephritic or nephrotic episode. It is the cumulative life-course risk created by kidney inflammation, chronic kidney disease (CKD), hypertension, cardiovascular morbidity, reduced nephron reserve, glucocorticoid toxicity, gonadal injury, impaired bone accrual, short stature, delayed puberty, school disruption, treatment fatigue, and loss of follow-up during transition [1,2].

This Viewpoint reframes the discussion around a practical principle: care for childhood-onset lupus nephritis should be evaluated not only by short-term proteinuria reduction, but by its ability to protect the child’s long-term health, development, and future. Modern treatment should be biopsy-driven, target-based, and toxicity-aware from the first visit. Pediatric clinicians should not wait until a child has accumulated irreversible kidney scarring or steroid-related damage before changing strategy. The best contemporary approach is early recognition, early biopsy when indicated, prompt control of active proliferative disease, rapid tapering of glucocorticoids when safe, maintenance long enough to prevent relapse, and support systems that make adherence realistic [1,2,3,4,5,6].

This perspective also requires a different way of reading guidelines. KDIGO, ACR, and EULAR provide essential structure, but pediatric clinicians must translate them through developmental reality [4,7,8,9]. A recommendation that is reasonable for an adult with stable work, autonomy, and completed growth may not carry the same trade-offs for a 13-year-old who is entering puberty, missing school, experiencing visible steroid toxicity, and depending on parents for medication administration. The question is not whether adult evidence should be used; it must be used because pediatric trials are limited. The question is how to use adult evidence without losing the child in front of us [10,11,12].

The phrase efficacy without toxicity is not a slogan for undertreatment. It means matching treatment intensity to histology and risk while reducing avoidable harm. A child with highly active proliferative disease needs rapid immunologic control. The same child also needs growth, fertility, bone health, mental health, and education protected. This dual obligation is what makes pediatric lupus nephritis different from simply applying adult protocols by body weight [10,11].

## 2. Classification and Kidney Biopsy

**Figure 1:**
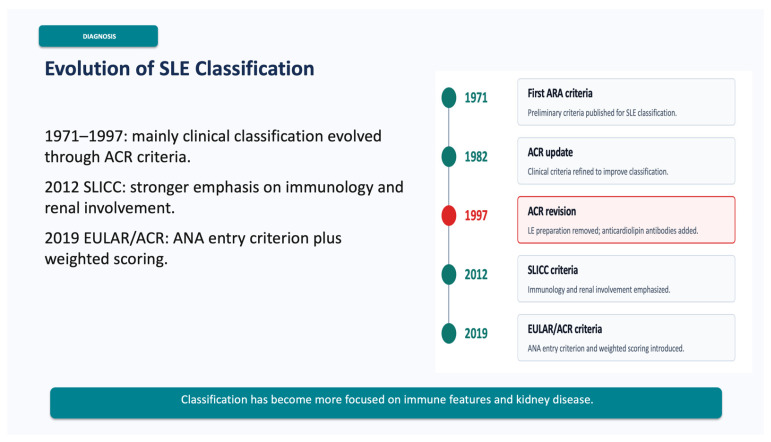
Evolution of SLE classification criteria from 1971 to 2019. The trend has moved from mainly clinical classification toward greater emphasis on immunology, renal involvement, and weighted scoring Abbreviation: ARA: American rheumatology association, ACR: American college of rheumatology, SLICC: Systemic lupus international collaborating clinics, EULAR: European League Against Rheumatism.

Modern SLE classification has evolved substantially, from mainly clinical criteria to systems that emphasize immunology and kidney disease (Figure 1). The 2012 SLICC criteria incorporated immunologic features and allowed biopsy-proven lupus nephritis with positive antinuclear or anti-dsDNA antibodies to classify SLE [12]. The 2019 EULAR/ACR criteria introduced a positive antinuclear antibody as an entry requirement, followed by weighted clinical and immunologic domains [7].

These criteria help frame diagnosis and research cohorts, but they do not define the kidney lesion. In a child with suspected nephritis, classification may tell us that SLE is likely; biopsy tells us what is happening inside the kidney.

**Figure 2:**
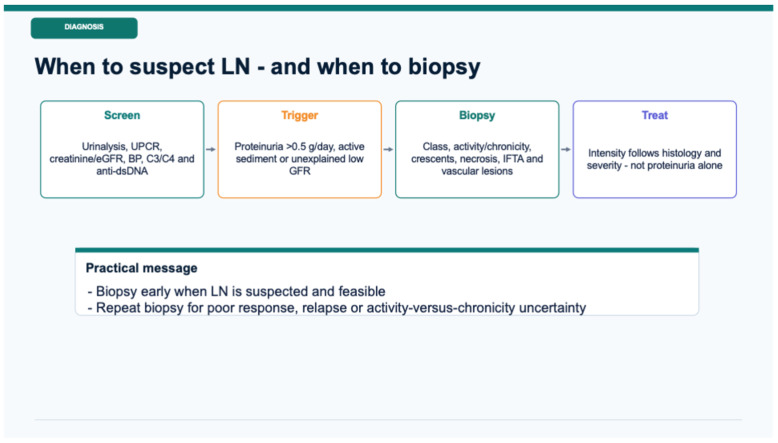
When to suspect LN—and when to biopsy.

This distinction matters because proteinuria alone can mislead. Clinical features may underestimate proliferative lesions, overestimate active inflammation when chronic scarring predominates, or miss vascular lesions such as thrombotic microangiopathy. The Saudi childhood lupus nephritis consensus recommends renal evaluation in newly diagnosed or suspected childhood SLE and highlights glomerular hematuria, proteinuria, unexplained creatinine elevation, hypoalbuminemia, hypertension, and active urinary sediment as prompts for nephritis assessment [2]. It also emphasizes biopsy as the most reliable diagnostic and prognostic marker, particularly when proteinuria exceeds 0.5 g/day, urine protein-creatinine ratio exceeds 50 mg/mmol, or eGFR declines without another explanation [2] (Figure 2).

Furthermore, Early diagnosis of lupus nephritis (LN) is expected to improve renal outcomes by enabling timely immunosuppressive therapy before irreversible damage. While pediatric comparative studies are limited, observational data consistently show that impaired kidney function at presentation, severe proteinuria, and hypertension predict poor prognosis [11,13,14].

Adult studies further reveal that even mild proteinuria may mask significant histologic disease, highlighting the need for early recognition. Maintaining vigilance, performing timely biopsy, and initiating therapy early increase the chances of remission and long-term kidney preservation [15].

The 2018 ISN/RPS revision strengthened the practical value of biopsy by clarifying lesion definitions, removing class IV-S and IV-G subdivisions, replacing the older active/chronic designation with modified NIH activity and chronicity indices, and introducing tubulointerstitial cutoffs [16]. For pediatric clinicians, a pathology report that states only class III or class IV is incomplete. Activity index, chronicity index, tubulointerstitial injury, and vascular lesions shape prognosis, treatment urgency, and the logic of escalation. Class switching also occurs, so a major change in proteinuria, eGFR, or urinary sediment should prompt reassessment and sometimes repeat biopsy [16].

## 3. Key cLN Treatment Milestones

The most important conceptual change in cLN is the movement from a two-phase induction-maintenance mindset toward continuous treat-to-target care [7,8,9]. Induction and maintenance remain useful labels, but the child and family experience treatment as one long course. The clinician’s task is to control inflammation rapidly, prevent relapse, reduce treatment burden, and keep the child engaged through adolescence and transition. The endpoint is not merely remission written in the chart; it is durable kidney response achieved early enough to prevent irreversible nephron loss [4,7,9].

**Figure 3:**
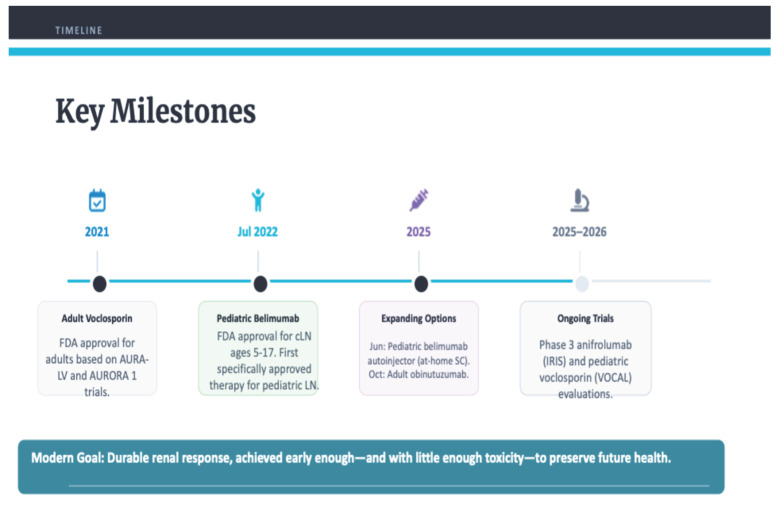
Key therapeutic and regulatory milestones in contemporary lupus nephritis care, emphasizing adult voclosporin approval, pediatric belimumab approval, the pediatric belimumab autoinjector, adult obinutuzumab approval, and ongoing/updated trial pathways.

These milestones should be interpreted through a pediatric lens: adult approvals expand therapeutic options, but pediatric use still requires attention to developmental toxicity, adherence, family burden, and the limited availability of child-specific randomized evidence (Figure 3).

**Figure 4:**
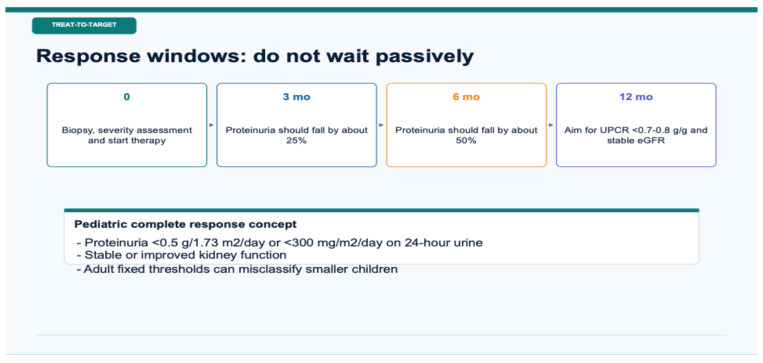
Treat-to-target response assessment in children: pediatric response definitions should use body-size-adjusted proteinuria thresholds together with stable or improved kidney function.

Proteinuria reduction has become a practical marker of whether the child is moving in the right direction. Pediatric consensus targets include at least 25% reduction in proteinuria by 3 months, at least 50% reduction by 6 months, and proteinuria below 0.7 g/day by 12 months [2]. These targets are not rigid substitutes for judgment, but they are clinically useful milestones. Waiting passively for 12 months in a child with a poor early trajectory risks allowing active inflammation to become chronic damage. Conversely, escalating therapy without checking adherence, drug exposure, and biopsy context may expose a child to toxicity without solving the true problem [1,2,5] (Figure 4).

In our view, every cLN visit should ask three questions. Is immune activity adequately controlled? Is kidney protection optimized? Is the treatment plan sustainable for this child and family? A regimen that is effective pharmacologically but impossible for an adolescent to follow is not a safe regimen. A low-toxicity regimen that leaves proliferative nephritis active is not safe either. Treat-to-target care must therefore integrate laboratory response, histology, medication exposure, adverse effects, adherence, and developmental context [17,18].

### 3.1. From High-Dose Steroids to Planned Glucocorticoid Minimization (Figure 5)

Historically, severe lupus nephritis was treated with high-dose glucocorticoids and cyclophosphamide. These regimens were kidney-saving, but their toxicities are especially consequential in children: infection, infertility, ovarian failure, avascular necrosis, osteoporosis, obesity, diabetes, short stature, cataract, mood disturbance, cosmetic changes, and treatment disengagement [1,2,10]. Steroid toxicity is not simply a cosmetic or quality-of-life issue. In adolescence, weight gain, acne, mood lability, and Cushingoid appearance can directly worsen adherence, and poor adherence can precipitate flare [1,2,10].

Contemporary guidelines increasingly make steroid minimization part of the initial plan. The ACR 2024 lupus nephritis guidance recommends intravenous methylprednisolone pulses followed by oral glucocorticoids at no more than 0.5 mg/kg/day, aiming for 5 mg/day or less by 6 months [5]. KDIGO similarly supports reduced-dose glucocorticoid regimens after short methylprednisolone pulses when kidney and extrarenal disease are improving [3,4]. This approach should be interpreted carefully. Steroid minimization is not steroid avoidance. In severe proliferative cLN, early pulses may be crucial. The change is that prolonged high-dose exposure should no longer be the default when safer tapering is possible.

The pediatric case for steroid minimization is particularly strong because the harms accumulate during development. Lower cumulative exposure may protect growth velocity, pubertal progression, bone mineralization, metabolic health, self-image, and adherence. The practical question is not whether steroids are useful; they are. The question is how to use them intensely enough to prevent nephron loss and briefly enough to avoid creating a second chronic illness through treatment toxicity [1,3,4,5,10].

**Figure 5:**
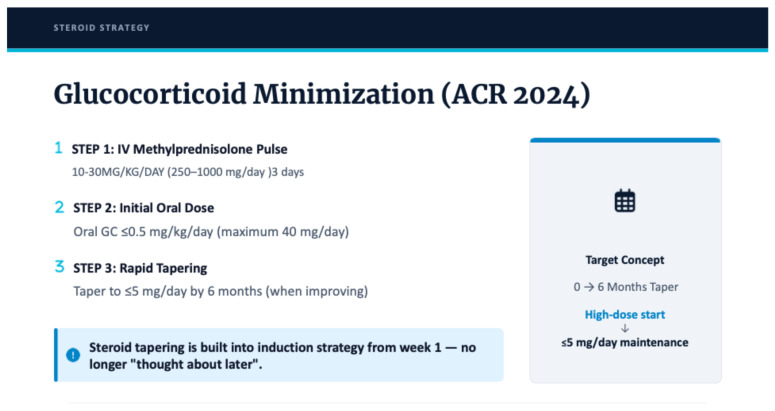
Planned glucocorticoid minimization strategy.

### 3.2. Mycophenolate as the Pediatric Backbone

**Figure 6:**
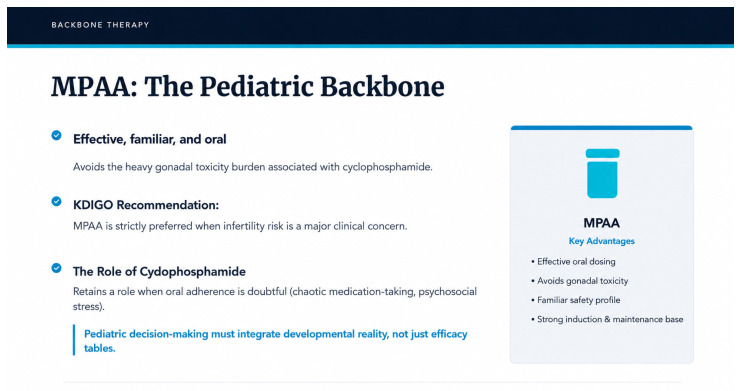
Mycophenolic acid analogues as the pediatric therapeutic backbone, with cyclophosphamide reserved for selected severe disease or situations in which adherence to oral therapy is doubtful.

Mycophenolic acid analogues, especially mycophenolate mofetil (MMF), remain central to pediatric proliferative lupus nephritis. They are effective, familiar, orally administered, and avoid the gonadal toxicity associated with cyclophosphamide. Pediatric consensus supports high-dose corticosteroids plus MMF as a preferred induction approach in many children with class III or IV disease, while intravenous cyclophosphamide remains an important alternative for severe presentations, including crescentic lesions or low eGFR [1,2,9] (Figure 6).

However, a viewpoint article should resist reducing modern care to MMF versus cyclophosphamide. The correct choice depends on histologic activity, chronicity, vascular lesions, proteinuria burden, kidney function, extrarenal disease, fertility risk, infection risk, family capacity, and adherence. In adolescents with chaotic medication-taking, supervised intravenous cyclophosphamide may sometimes be safer than an oral regimen that is rarely taken. Conversely, in a child at high risk of gonadal toxicity and with supportive adherence structures, MMF-centered therapy may better align with long-term developmental goals [1,2,3,4,9].

The rise of triple therapy does not displace MMF; it often builds on it. KDIGO 2024 recommends glucocorticoids plus one of several initial options for active class III or IV lupus nephritis: mycophenolic acid analogues, low-dose intravenous cyclophosphamide, belimumab plus mycophenolic acid analogues or low-dose cyclophosphamide, or mycophenolic acid analogues plus a calcineurin inhibitor [3,4]. This menu should not be read as a mandate to use the newest drug. It should be read as permission to individualize early, rather than waiting for failure in a child who is predictably high risk.

### 3.3. Belimumab as the Most Mature Pediatric Biologic Option

**Figure 7:**
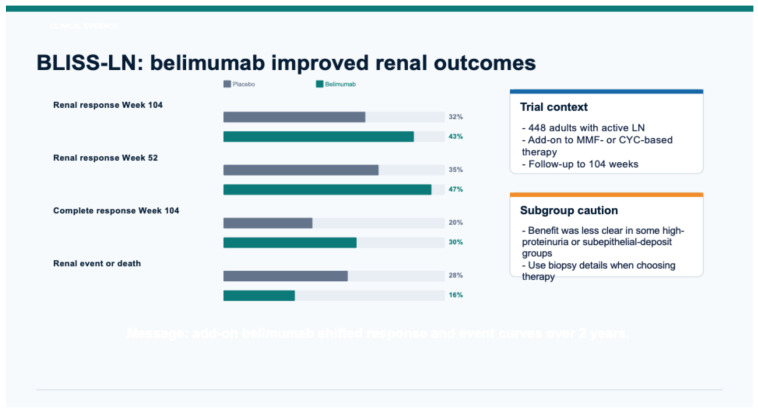
In adults, the BLISS-LN trial showed that belimumab added to standard therapy improved kidney response over 104 weeks with permission from Ref. [19].

Belimumab has become the biologic most relevant to pediatric lupus nephritis because it now has pediatric regulatory and practical relevance. In adults, the BLISS-LN trial showed that belimumab added to standard therapy improved kidney response over 104 weeks [8] (Figure 7), and subsequent analyses suggested kidney-protective benefit [19]. For children, the more important milestone is that belimumab was approved for lupus nephritis in patients aged 5–17 years in 2022, followed in 2025 by approval of the 200 mg/mL autoinjector for subcutaneous use in children aged 5 years and older with active lupus nephritis receiving standard therapy [19,20,21,22].

The autoinjector matters because the treatment route matters. Pediatric chronic disease care is not only pharmacology. It is school attendance, transport, family workload, needle anxiety, infusion logistics, and adolescent autonomy. An at-home subcutaneous option may reduce treatment burden and support continuity. That does not prove superiority over other strategies, but it makes belimumab easier to integrate into real pediatric life.

Child-specific outcome data remain limited. A pediatric study has reported efficacy and safety of belimumab combined with standard therapy in childhood lupus nephritis, but randomized pediatric evidence remains much thinner than adult evidence [22,23]. Therefore, belimumab should be viewed as the most mature pediatric biologic option, not as a universal add-on. It is most persuasive in relapse-prone disease, prominent extrarenal activity, or situations where steroid-sparing is especially important [22,23].

### 3.4. Calcineurin Inhibitors and Voclosporin

**Figure 8:**
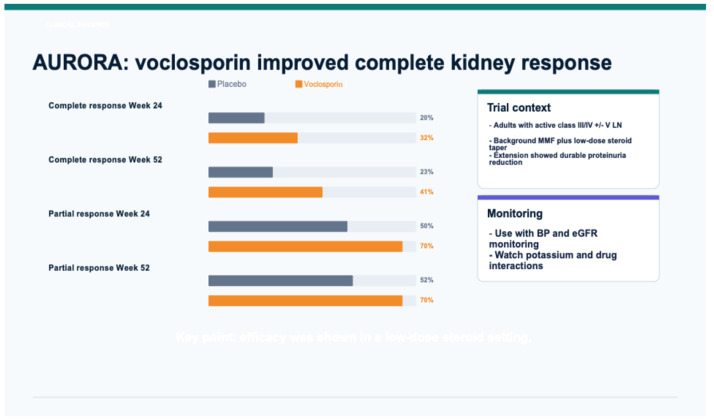
In adults, the AURORA trial showed that the addition of Voclosporin improved renal response when added to MMF and rapidly tapered glucocorticoids with permission from Ref. [23].

Calcineurin-inhibitor (CNI)-based triple therapy is attractive in selected patients because CNIs can reduce proteinuria rapidly, particularly when podocyte injury contributes to nephrotic-range proteinuria. KDIGO notes that CNI-containing regimens may be especially useful when kidney function is relatively preserved and proteinuria is heavy [3,4]. Voclosporin is the best-studied modern CNI in lupus nephritis, supported in adults by AURA-LV and AURORA 1, where it improved kidney response when added to MMF and rapidly tapered glucocorticoids [24,25] (Figure 8).

For children, the limitation is straightforward: adult efficacy cannot be assumed to equal pediatric certainty. Voclosporin remains far better established in adults than in children. The VOCAL pediatric/adolescent study (NCT05288855) was designed to assess kidney response after 24 weeks in children and adolescents with active lupus nephritis, but the current Clinical Trials.gov record lists the study as terminated, with last update posted 10 February 2026 [26]. There are no further details available online on why this study was terminated. Possible reasons include inability to recruit enough patients, sponsor’s decision or changes in development strategies, administrative or financial reasons, or safety concerns, which is unlikely given that no such explanation was mentioned. The termination of the VOCAL study is disappointing because it leaves an important evidence gap. This distinction is important. A pediatric viewpoint should not present voclosporin as standard child-specific evidence when the evidence base remains incomplete.

Nevertheless, voclosporin and other CNIs remain conceptually important. In a child with preserved GFR, severe proteinuria, and a need for rapid antiproteinuric effect, CNI-based therapy may be reasonable in expert hands. But it should be framed as individualized, often off-label pediatric use, requiring careful monitoring for nephrotoxicity, hypertension, drug interactions, and long-term safety [3,4,26].

### 3.5. Pure Class V Disease

Pure class V lupus nephritis deserves separate thinking. Its biology, risks, and treatment logic differ from proliferative disease. Heavy proteinuria in class V disease may drive edema, dyslipidemia, thrombosis risk, infection risk, and cardiovascular morbidity, even when proliferative inflammation is absent [3,4]. At the same time, the evidence base for pure class V disease is thinner than for class III/IV disease, and aggressive immunosuppression should not be automatic.

In our view, class V treatment should be guided by proteinuria burden, kidney function, thrombotic risk, systemic lupus activity, biopsy chronicity, and patient-specific toxicity considerations. CNIs may be particularly useful when nephrotic-range proteinuria dominates, and adult AURORA data suggest faster proteinuria reduction in some membranous subgroups [24,25]. Belimumab may be less compelling in isolated nephrotic-range class V disease than in proliferative or mixed disease, although it may still be appropriate when systemic activity or relapse risk is prominent [3,4,19].

For children, the practical message is simple: do not undertreat heavy membranous disease, but do not automatically treat it like proliferative nephritis. The biopsy, proteinuria burden, thrombotic risk, eGFR, and lupus phenotype should determine intensity.

## 4. Maintenance, De-Escalation, and Adolescence

Maintenance therapy is where pediatric nephritis care either succeeds or fails. KDIGO recommends mycophenolic acid analogues for maintenance after initial therapy and identifies azathioprine as an alternative when mycophenolate is not tolerated, unavailable, or pregnancy is being considered [3,4]. KDIGO also suggests that total immunosuppression plus maintenance for proliferative lupus nephritis should generally continue for at least 36 months, while ACR guidance broadly supports total therapy for at least 3–5 years after complete kidney response [3,4,5].

This longer horizon is appropriate in children because premature withdrawal can turn one flare into repeated flares across adolescence and young adulthood. Repeated lupus nephritis flares can cause cumulative nephron loss, CKD, kidney failure, and more steroid exposure [1]. Yet prolonged therapy also has costs: infection risk, teratogenicity counseling, medication fatigue, family conflict, and adolescent desire for normality. The best maintenance regimen is not necessarily the smallest regimen; it is the smallest effective regimen that the child and family can realistically sustain.

De-escalation should therefore be deliberate. It should follow stable clinical response, optimized supportive care, evidence of adherence, and sometimes repeat biopsy when the clinical picture is ambiguous. It should not occur simply because the child looks well or because the family is exhausted. Conversely, indefinite treatment without a plan can erode trust and adherence. Pediatric teams should name the maintenance goal, explain the expected duration, revisit fertility and pregnancy prevention when relevant, and prepare for transition long before transfer to adult care [1,3,4,5].

**Figure 9:**
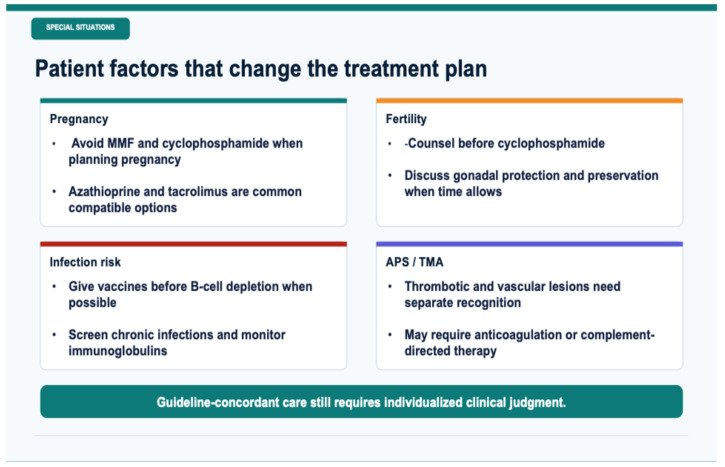
Patient factors that change the treatment plan of lupus nephritis. Abbreviations: ASP: anti-phospholipid syndrome, TMA: thrombotic microangiopathy.

Patient-specific factors that may change the lupus nephritis treatment plan. In pregnancy or planned pregnancy, MMF and cyclophosphamide should be avoided, while azathioprine and tacrolimus are commonly compatible options. Before cyclophosphamide, fertility counseling and preservation should be discussed. Infection risk should be reduced by giving vaccines before B-cell–depleting therapy when possible, screening for chronic infections, and monitoring immunoglobulin levels. Patients with APS or TMA require special attention because thrombotic or vascular lesions may need additional treatment, such as anticoagulation or complement-directed therapy. Overall, even guideline-based care must be individualized for each patient (Figure 9).

## 5. Refractory Disease and Treatment Exposure

A useful development in recent guidance is the explicit insistence that inadequate response should trigger assessment of medication dose, exposure, and adherence before disease is labeled biologically refractory [5]. This is particularly important in adolescents. Nonadherence can mimic drug failure, and escalating therapy without addressing adherence can add toxicity without improving kidney outcomes. The first question in apparent refractory disease is not always which biologic to add. It is whether the child received the treatment already prescribed.

Once inadequate exposure has been excluded, escalation from dual therapy to triple therapy, switching between triple regimens, adding anti-CD20 therapy, or referral for investigational therapy may be appropriate [3,4,5,6]. Repeat biopsy can be decisive. Persistent proteinuria may reflect active immune injury, chronic scarring, residual membranous disease, thrombotic microangiopathy, or noninflammatory CKD. These scenarios require different treatments, and in children, both undertreatment and overtreatment carry lifelong consequences [1,2].

Renal histology itself does not necessarily track clinical remission, and this distinction is particularly important in children, in whom repeated invasive procedures must be justified. In adults with proliferative lupus nephritis who underwent protocol re-biopsy after induction, activity indices improved overall, but only half of complete clinical responders had an activity index of 3 or less, nearly one-third still had high histologic activity, and 62% of those with complete histologic remission still had persistent clinical kidney activity, mainly proteinuria [27].

Pediatric data tell a similar story. In a cohort of children with proliferative lupus nephritis who underwent surveillance biopsy approximately one year after diagnosis, activity scores improved in both complete and incomplete clinical responders, yet more than half of the children classified as being in complete kidney response still had persistent proliferative nephritis on the repeat biopsy, and chronicity scores worsened in both groups [28]. A separate pediatric series of repeat biopsies performed mainly for relapsing proteinuria found that nearly half of children showed a change in histologic class between biopsies, and that glomerulosclerosis and tubular atrophy increased significantly even though short-term clinical remission rates were similar regardless of class change [29]. Taken together, these data argue against relying on laboratory remission alone to judge histologic response or to guide de-escalation. A repeat biopsy, when clinically indicated, can uncover ongoing proliferative activity or accumulating chronicity that laboratory parameters miss, and in pediatric series, it directly changed the treatment plan in a meaningful proportion of children, whether by escalating, de-escalating, or holding therapy steady [28,30].

This is where the viewpoint of efficacy without toxicity becomes most concrete. It is not enough to intensify. We must intensify the right treatment for the right pathology and the right reason.

## 6. New Adult Therapies and Pediatric Evidence Gaps

**Figure 10:**
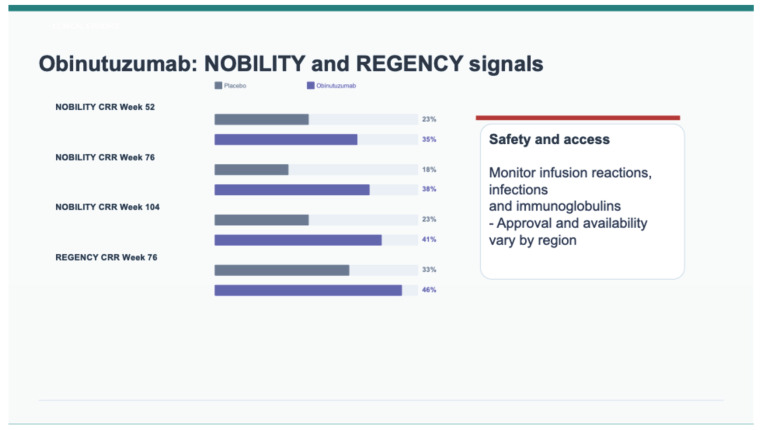
The NOBILITY trial showed improved complete renal response when obinutuzumab was added to MMF and glucocorticoids in proliferative lupus nephritis with permission from Ref. [29].

Obinutuzumab and anifrolumab illustrate the opportunity and risk of translating adult innovation to pediatrics. Obinutuzumab has become an important adult update. The NOBILITY trial showed improved complete kidney response when obinutuzumab was added to MMF and glucocorticoids in proliferative lupus nephritis [31]. Subsequent phase 3 REGENCY results supported adult regulatory approval, with Roche reporting complete renal response in 46.4% of patients receiving obinutuzumab plus standard therapy compared with 33.1% receiving standard therapy alone [31,32] (Figure 10). European approval followed for adults with active class III or IV lupus nephritis, with or without class V features [32].

For pediatric clinicians, obinutuzumab is relevant because it validates deeper B-cell targeting as a kidney strategy and may shape future pediatric studies. But it remains an adult-approved therapy. Child-specific dosing, infection risk, immunoglobulin effects, vaccine response, long-term safety, and sequencing require pediatric data before routine adoption.

Anifrolumab points toward another future direction: interferon pathway targeting. Phase 2 TULIP-LN data suggested numerically higher kidney response with intensified anifrolumab in adults with active proliferative lupus nephritis, and the phase 3 IRIS trial remains active but not recruiting, with estimated primary completion in 2027 [33,34]. For children, anifrolumab is currently conceptual rather than routine. It reminds us that future cLN care may be biomarker-directed, but pediatric evidence must catch up before precision therapy becomes more than aspiration Table 1.

## 7. Immediate Changes for Pediatric Practice

Several changes can be implemented now without waiting for perfect pediatric trials. First, biopsy should be pursued early when nephritis is suspected and should be repeated when the clinical course is discordant with expectations. Second, steroid tapering should be planned from the beginning rather than improvised months later. Third, hydroxychloroquine, blood pressure control, RAAS blockade when indicated, infection prevention, bone protection, fertility counseling, and vaccination planning should be considered part of treatment, not background tasks [1,2,3,4,5].

Fourth, adherence should be assessed routinely and nonjudgmentally. Adolescents rarely benefit from being told simply to take medications. They need simplified regimens, shared decision-making, family support, mental health screening, school-aware scheduling, and adult-transition preparation. Fifth, new therapies should be used thoughtfully. Belimumab has the strongest pediatric footing among biologic options; CNI-based strategies may be useful for selected proteinuric phenotypes; adult obinutuzumab and anifrolumab data should stimulate pediatric trials rather than premature generalization [8,9,10,11,12,13,14,15,16,17,18,19,20,21,22,23,24,25,26,27,28,29,30,31,32,33,34,35].

Finally, pediatric trials must measure outcomes that children live with: growth, puberty, bone health, fertility, school participation, treatment burden, adherence, transition success, and long-term kidney survival. If trials measure only short-term renal response, they will miss what makes childhood disease unique.

## 8. Adherence, Transition, and Pediatric Outcomes

Pediatric lupus nephritis is often described through pathology classes and response definitions, but the events that decide long-term outcomes frequently occur outside the biopsy report. Adolescents may skip medications because of steroid-related appearance changes, school schedules, nausea, pill burden, needle fatigue, depression, family conflict, financial barriers, or a belief that treatment is no longer needed once edema resolves. Nonadherence is not a moral failure; it is a predictable feature of chronic disease during adolescence and must be managed as actively as hypertension or proteinuria [1].

Cohort data accumulated over the past several years reinforce how long-term outcome depends on early treatment response and disease trajectory, more so than on histologic class assigned at diagnosis alone. A 20-year Hong Kong cohort of children with biopsy-proven cLN reported favorable long-term kidney survival overall, but a composite outcome of advanced CKD, KF, or death occurred in a meaningful minority by 15 years, driven mainly by need for dialysis at presentation, non-response at 12 months, and repeated kidney flares [36].

A 13-center Korean cohort of 216 children similarly reported advanced CKD in 14.8% after a mean follow-up of 7.8 years. Failure to achieve remission at 12 months and male sex were independently associated with progression to advanced CKD, whereas proliferative histology was not an independent predictor in this cohort [37]. A Turkish single-center cohort spanning two decades evaluated the short- and long-term renal outcomes of children with lupus nephritis [38]. Complementing these findings, adults followed for approximately two decades after childhood-onset disease continued to accumulate both kidney and extrarenal damage well into adulthood, underscoring that the consequences of cLN extend far beyond the transition to adult care [39]. An editorial summarizing a large Indian pediatric cohort reached a consistent conclusion: multiple kidney flares and infections were the dominant long-term morbidities, with rapidly progressive glomerulonephritis, treatment non-response, and KF emerging as the strongest predictors of adverse kidney outcome [8]. These cohorts, together with their key predictors of poor long-term outcome, are summarized in Table 2.

In practical terms, adherence should be measured and discussed repeatedly. Pharmacy refill patterns, drug levels when available, direct questions asked without blame, and family-based problem solving can reveal barriers before relapse occurs. When a child appears refractory, the first step should be to determine whether the medication was taken at an adequate dose and duration. This approach protects the child from unnecessary escalation while also protecting the kidney from unrecognized undertreatment [1,5].

Transition from pediatric to adult care is another therapeutic intervention. Transfer should not occur as an administrative handover at age eighteen. It should be a staged process in which the young person learns the diagnosis, medication names, reproductive risks, warning symptoms, appointment skills, insurance or access issues, and how to seek urgent help. The receiving adult team should understand the pediatric disease course, biopsy history, cumulative cyclophosphamide or steroid exposure, adherence barriers, fertility counseling already provided, and the family context. A failed transition can undo years of excellent pediatric care [1].

For this reason, future pediatric trials and registries should not limit success to proteinuria and eGFR. They should also capture growth velocity, pubertal development, school attendance, fatigue, mental health, fertility preservation, patient-reported treatment burden, medication persistence, and successful transfer to adult care. These outcomes are not peripheral. They are the outcomes that determine whether a child with cLN becomes an adult with health, autonomy, and preserved kidney function.

## 9. Operationalizing Efficacy Without Toxicity

A practical clinic model for cLN should begin with shared risk stratification. Histology defines the kidney lesion, but the management plan should also document proteinuria burden, eGFR, blood pressure, complements, anti-dsDNA antibodies, extrarenal disease, infection risk, vaccine status, fertility concerns, growth and pubertal status, mental health, school needs, family support, and medication access. These domains should not be buried in narrative notes; they should be visible in the treatment plan because they determine whether the regimen is safe and sustainable [1,2,3,4,5,6].

Second, every induction plan should include a steroid exit strategy. The plan should specify pulse dosing, starting oral dose, expected taper, target by 3 and 6 months, and toxicity monitoring. When tapering fails, clinicians should ask why: persistent immune activity, inadequate adherence, unresolved nephrotic proteinuria, insufficient background therapy, or fear-driven inertia. Naming the reason prevents prolonged steroid exposure from becoming the default.

Third, nephroprotection should be prescribed with the same seriousness as immunosuppression. Blood pressure targets, RAAS blockade when appropriate, salt counseling, vitamin D and bone protection, sun protection, infection prevention, and reproductive counseling are not optional additions. They modify the long-term kidney and developmental trajectory. A child whose immune activity is controlled but whose hypertension, bone health, or transition plan is neglected has not received complete lupus nephritis care [1,2,3,4,5].

Finally, escalation should be phenotype-based rather than trend-based alone. Heavy proteinuria with preserved GFR may suggest a role for CNI-based therapy; relapse-prone multisystem disease may favor belimumab; persistent activity after adequate exposure may justify anti-CD20 strategies or trial referral; chronic scarring should shift attention toward nephroprotection rather than endless immunosuppression. This is the practical meaning of precision care in cLN today: not a perfect biomarker, but a disciplined integration of biopsy, trajectory, exposure, toxicity, and pediatric context.

## 10. Limitations and Perspectives

This viewpoint has several important limitations. First, it is a narrative opinion article rather than a systematic review or formal guideline. Much of the therapeutic reasoning is based on extrapolation from adult lupus nephritis studies because pediatric randomized data remain limited. This is particularly relevant for newer or emerging treatments, including voclosporin, obinutuzumab, anifrolumab, JAK/STAT inhibitors, BAFF/APRIL-targeted therapies, co-stimulation blockade, and complement inhibitors. Therefore, adult advances should guide pediatric research and inform selected clinical decisions, but they should not be adopted uncritically as routine pediatric standards.

Second, most available evidence still focuses mainly on kidney response, especially proteinuria reduction and preservation of kidney function. These outcomes are essential, but they do not fully capture the burden of childhood-onset lupus nephritis. In children, treatment decisions must also consider growth, puberty, fertility, bone health, steroid toxicity, infection risk, school participation, adherence, family burden, and transition to adult care.

**Figure 11:**
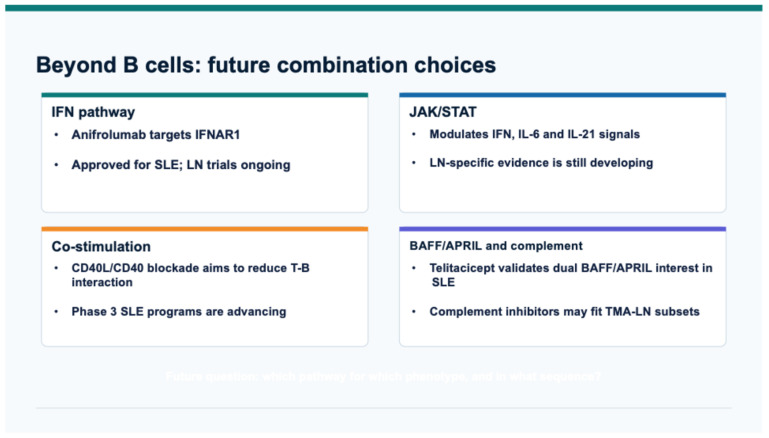
Future question: which pathway for which phenotype, and in what sequence.

Figure 11 highlights the future therapeutic landscape beyond B-cell depletion. Potential combination strategies may target the interferon pathway, JAK/STAT signaling, T-cell co-stimulation, BAFF/APRIL biology, or complement activation. However, the key unanswered question is which pathway should be selected for which phenotype, at what stage of disease, and in what treatment sequence. For example, interferon-pathway inhibition may be relevant in patients with strong systemic lupus activity, whereas complement-directed therapy may be more appropriate for selected patients with thrombotic microangiopathy or vascular lesions.

Future work should prioritize pediatric-specific clinical trials, long-term registries, and outcome measures that combine kidney survival with child-centered outcomes. Research should define which children benefit most from belimumab, CNI-based therapy, anti-CD20 treatment, interferon-pathway inhibition, or other emerging targeted therapies, while also determining how these agents can reduce cumulative glucocorticoid exposure.

Until stronger pediatric evidence becomes available, treatment should remain biopsy-driven, target-based, and individualized. The goal is not only remission, but durable renal control with the lowest feasible toxicity, allowing children with lupus nephritis to reach adulthood with preserved kidney function, growth, fertility, and long-term treatment engagement.

## 11. Conclusions

The latest update in pediatric lupus nephritis is not a single drug approval; it is a change in treatment logic. Contemporary cLN care should be biopsy-driven, target-based, and designed to achieve early renal control with the lowest feasible cumulative toxicity. Mycophenolic acid analogues remain the major therapeutic backbone. Glucocorticoids are still essential, but prolonged high-dose exposure should no longer be accepted as inevitable. Triple therapy is increasingly part of mainstream first-line thinking in selected patients, especially with belimumab or CNI-based combinations, while newer adult therapies should be viewed as signals for pediatric research rather than automatic pediatric standards.

Pure class V disease requires phenotype-guided treatment. Maintenance therapy must be long enough to prevent relapse but sustainable enough for adolescence. Refractory disease management must be honest about adherence and must use repeat biopsy when needed. Supportive care is not secondary; it is treatment. The child with lupus nephritis should not only survive the flare, but also should enter adulthood with preserved kidney function, protected growth and fertility, and a treatment history that minimized rather than compounded lifelong risk. That is the real meaning of efficacy without toxicity. Table 3 shows the practical approach to common clinical scenarios in childhood lupus nephritis.

## Figures and Tables

**Table 1:** Pediatric approval status and evidence for selected biologics in systemic lupus erythematosus and lupus nephritis.

Medicine	Target	Pediatric Approval Status	Pediatric Evidence
Belimumab	B-cell activating factor (BAFF) inhibitor	Approved for children ≥ 5 years with active systemic lupus erythematosus (SLE).	PLUTO trial (NCT01649765) demonstrated efficacy and safety in pediatric SLE. Pediatric lupus nephritis (LN)-specific evidence remains limited and is largely extrapolated from adult BLISS-LN data [20,21,22,23].
Obinutuzumab	Type II anti-CD20 B-cell-depleting monoclonal antibody	Not approved for pediatric SLE or LN.	Pediatric LN trial ongoing: POSTERITY (NCT05039619). Current evidence is limited to adult trials and pediatric case reports/series [31,32].
Anifrolumab	Type I interferon receptor antagonist	Not approved for pediatric SLE or LN.	Pediatric SLE trial ongoing: BLOSSOM (NCT05835310). Pediatric LN-specific data are currently unavailable [33,34].

Abbreviations: BAFF, B-cell activating factor; NCT, ClinicalTrials.gov identifier.

**Table 2:** Selected contemporary cohorts reporting long-term outcomes of childhood-onset lupus nephritis.

Study (Region, Year)	Cohort and Follow-Up	Long-Term Outcome	Key Predictors of Poor Outcome
Chan et al. [34], Hong Kong	*n* = 92 children; median follow-up 10.3 y (range to 20 y)	Kidney survival 94.6% at 10 y; 83.2% free of advanced CKD/ESKD/death at 15–20 y	Acute dialysis at presentation, non-response at 12 months, repeated renal flares
Park et al. [36], South Korea (13 centers)	*n* = 216 children; mean follow-up 7.8 y	Advanced CKD in 14.8% at last follow-up	Male sex and poor treatment response (failure to achieve remission at 12 months)
Demir et al. [37], Turkey	*n* = 53 children (of 102 with childhood-onset SLE); single academic center, 2000–2020	Short-term remission and favorable 5- and 10-year renal survival	Dialysis at presentation, male sex, and failure to achieve remission
Groot et al. [39], adults followed after childhood-onset SLE	Multi-decade cohort of adults with childhood-onset disease	Continued accrual of renal and extra-renal organ damage	Cumulative disease burden and treatment-related damage
Lama MV et al. [40], an Indian cohort	*n* = 75 children, Indi, 2010–2024	Kidney flares and infections were the dominant long-term morbidities	Rapidly progressive glomerulonephritis, treatment non-response, severe kidney flare

Abbreviations: CKD, chronic kidney disease; ESKD, end-stage kidney disease; SLE, systemic lupus erythematosus.

**Table 3:** Practical approach to common clinical scenarios in childhood lupus nephritis.

Clinical Question	Current Approach	Viewpoint Rationale
Suspected cLN	Screen urine, kidney function, serology; biopsy early when indicated.	Histology defines class, activity, chronicity, vascular lesions, prognosis, and therapy [1,2].
Active class III/IV +/− V	Glucocorticoids plus MPAA,/low-dose cyclophosphamide, belimumab-based, or CNI-based therapy in selected patients.	Combination therapy can improve early control while enabling steroid minimization [3,4,5,6].
Pure class V	Proteinuria- and phenotype-guided therapy; consider MMF and/or CNI when nephrotic proteinuria is prominent.	Biology and risk profile differ from proliferative disease [3,4].
Maintenance	MPAA commonly preferred; continue long enough to prevent relapse; taper steroids as low as possible. May continue triple therapy.	Repeated flares drive nephron loss and steroid toxicity [1,3,4,5].
Inadequate response	Check adherence and drug exposure first; consider repeat biopsy and regimen switch or escalation.	Persistent proteinuria may represent activity, chronicity, membranous disease, TMA, or CKD [1,2,5].

Abbreviations: cLN, childhood-onset lupus nephritis; MPAA, mycophenolic acid analogues; MMF, mycophenolate mofetil; CNI, calcineurin inhibitor; TMA, thrombotic microangiopathy; CKD, chronic kidney disease.

## Data Availability

No new data were created or analyzed in this study. Data sharing is not applicable to this article.
